# Fetal mortality before and during the COVID-19 pandemic: a single-center cohort study in Rio de Janeiro, Brazil

**DOI:** 10.1590/1806-9282.20250382

**Published:** 2025-07-07

**Authors:** Gustavo Yano Callado, Edward Araujo, Carolina Aquino Guedes Ramos, José Maria Andrade Lopes, Danielle Negri, Maria Elizabeth Lopes Moreira, Renato Augusto Moreira de Sá

**Affiliations:** 1 Hospital Israelita Albert Einstein, Faculdade Israelita de Ciências da Saúde Albert Einstein, – São Paulo (SP), Brazil.; 2 Universidade Federal de São Paulo, Escola Paulista de Medicina, Department of Obstetrics – São Paulo (SP), Brazil.; 3 Universidade Municipal de São Caetano do Sul, Discipline of Woman Health – São Caetano do Sul (SP), Brazil.; 4 Instituto Nacional de Saúde da Mulher, da Criança e do Adolescente Fernandes Figueira, Department of Fetal Medicine – Rio de Janeiro (RJ), Brazil.; 5 Maternidade Perinatal Rede D’Or, Department of Obstetrics, Gynecology, and Fetal Medicine – Rio de Janeiro (RJ), Brazil.; 6 Universidade Federal Fluminense, Department of Obstetrics – Niterói (RJ), Brazil.

**Keywords:** COVID-19, Fetal death, Maternal health services, Prenatal care, Health disparities

## Abstract

**OBJECTIVE:**

The aim of this study was to compare the characteristics of pregnancies that resulted in fetal death before and during the COVID-19 pandemic in a private maternity hospital in Rio de Janeiro, Brazil.

**METHODS:**

This retrospective cohort study considered all pregnancies that ended in fetal death in a private maternity hospital in Rio de Janeiro, Brazil, from January 2018 to December 2021. Maternal and fetal characteristics were compared between the periods before the pandemic and during the pandemic. Data were extracted from electronic medical records, and statistical analyses included Student's t-test, chi-square test, and Fisher's exact test where applicable.

**RESULTS:**

Among 41,162 deliveries at Casa de Saúde Perinatal, 88 (0.21%) resulted in fetal death, with 38 (43.2%) occurring pre-pandemic (2018–March 2020) and 50 (56.8%) during the pandemic (March 2020–2021). Maternal demographics, comorbidities, and prepartum complications showed no statistically significant differences between groups. COVID-19 infection was identified in 12% of the pandemic-group pregnancies, but no significant disparities in fetal death causes or abnormalities were found.

**CONCLUSIONS:**

No significant differences were observed in maternal or fetal characteristics, prepartum complications, or fetal mortality rates between the pre-pandemic and pandemic periods in this private hospital setting. However, this does not reflect the broader Brazilian healthcare reality, where disparities in access and resources may have influenced outcomes differently.

## INTRODUCTION

The COVID-19 pandemic has had varied effects on pregnancy outcomes, influencing obstetric care practices and maternal–fetal health differently across regions and healthcare systems. Studies have reported increased rates of preterm birth and ­cesarean delivery in COVID-19-positive pregnancies, but findings remain inconsistent across settings^1,2^. These variations underscore the impact of healthcare infrastructure, public health responses, and socioeconomic disparities on maternal and fetal health outcomes^3^.

Fetal death is a key indicator of maternal–fetal health and reflects the overall quality of healthcare services^4,5^. While some studies suggest increased fetal mortality during the pandemic due to healthcare system disruptions, others have found no change or even a decline in perinatal deaths^6^. Given these discrepancies, investigating how fetal mortality evolved during the pandemic in different healthcare settings is essential for understanding its broader implications.

The aim of this study was to compare the characteristics of pregnancies that resulted in fetal death before and during the COVID-19 pandemic in a private maternity hospital in Rio de Janeiro, Brazil, and to evaluate the impact of the pandemic on maternal–fetal health in a well-equipped center in a ­middle-income country.

## METHODS

### Population and setting

This retrospective cohort study included all pregnancies that ended in spontaneous fetal death that received medical care at the Casa de Saúde Perinatal (Barra and Laranjeiras units) in the city of Rio de Janeiro, Brazil, from January 2018 to December 2021. The total sample was considered, without restrictions regarding maternal age, gestational age (GA), or any other factors. Women included in this study received prenatal care in the private healthcare system and were referred to or sought the service after fetal death.

This study adhered to the Strengthening the Reporting of Observational Studies in Epidemiology (STROBE) guidelines. STROBE was used to structure the study design, data collection, and statistical analysis, ensuring methodological rigor and reproducibility.

Data were retrieved from electronic medical records, ensuring comprehensive case documentation. This study is part of a broader research initiative entitled Rede de Pesquisa em SARS-CoV-2/COVID-19 na Assistência Perinatal ("SARS-CoV-2/COVID-19 Research Network in Perinatal Care"), currently underway at the Instituto Fernandes Figueira (CAAE 30598020.0.0000.5269), and aligned with its specific objectives.

### Study groups

The patients were divided into two groups: before the pandemic and during the pandemic, based on the date of the fetal death. The date we chose for the division of the two groups was March 2020 when the World Health Organization and the governmental authorities in Rio de Janeiro began implementing measures to contain the infection.

For both groups, maternal and fetal characteristics were assessed. Maternal characteristics included age at fetal death, race, parity, comorbidities, body mass index (BMI), mode of delivery, prepartum complications, and COVID-19 infection. Fetal characteristics included GA at death, sex, twinning, probable cause of death, and abnormal findings on ultrasonographic examination.

### Statistical analysis

The groups before and during the pandemic were statistically compared across all reported characteristics. The Student's t-test was used for quantitative variables, while the chi-square test was applied to qualitative variables. When any variable had an occurrence ≤5 in either group, Fisher's exact test was used as a substitute. Analyses were conducted in R software version 4.3.3.

## RESULTS

Among 41.162 deliveries performed at Casa de Saúde Perinatal between the study period (21.330 between 2018 and 2019, and 19,832 between 2020 and 2021), 88 (0.21%) pregnancies resulted in fetal death. Of these, 38 (43.2%) occurred between January 2018 and March 2020 (pre-pandemic group), and 50 (56.8%) occurred between March 2020 and December 2021 (pandemic group).

The two groups exhibited similar maternal demographic and pregnancy-related characteristics. No significant differences were observed in maternal age, race, BMI, parity, mode of delivery, and recurrent miscarriage rates. When comorbidities were analyzed, no difference was seen in the rates of diabetes mellitus, chronic hypertension, gestational hypertension/preeclampsia, thrombophilia, or others. The maternal characteristics are summarized in Table 1.

**Table 1:** Maternal characteristics.

Variables		Pre-pandemia (n=38)	Pandemia (n=50)	p-value§
Age, mean years (SD)		33.9 (5.5)	34.0 (4.7)	0.929
Body mass index, mean kg/m^2^ (SD)*		29.5 (5.7)	28.7 (4.1)	0.539
Race				
	White, n (%)	26 (68.4)	30 (60.0)	0.555
	Black, n (%)	0 (0.0)	2 (4.0)	0.504
	Mixed, n (%)	10 (26.3)	17 (34.0)	0.589
	Asian, n (%)	2 (5.3)	1 (2.0)	0.576
Any comorbidity, n (%)		20 (52.6)	21 (42.0)	0.439
	Chronic hypertension, n (%)	3 (7.9)	2 (4.0)	0.648
	Diabetes mellitus, n (%)	3 (7.9)	1 (2.0)	0.311
	Gestational hypertension/preeclampsia, n (%)	6 (15.8)	6 (12.0)	0.842
	Thrombophilia, n (%)	4 (10.5)	1 (2.0)	0.161
	More than one comorbidity, n (%)	6 (15.8)	3 (6.0)	0.166
	Other comorbidities, n (%)	10 (26.3)	14 (28.0)	1.000
Parity				
	Primiparous, n (%)	13 (34.2)	25 (50.0)	0.206
	Secundiparous, n (%)	11 (28.9)	15 (30.0)	1.000
	Tertiparous or more, n (%)	14 (36.8)	10 (20.0)	0.131
	Recurrent miscarriages, n (%)	4 (10.5)	6 (12.0)	1.000
Type of delivery				
	Vaginal, n (%)	15 (39.5)	21 (42.0)	0.984
	Cesarean section, n (%)	21 (55.3)	26 (52.0)	0.930
	Curettage, n (%)	2 (5.3)	3 (6.0)	1.000

* Data from 31 patients from the pre-pandemia group and 27 patients from the pandemia group.

§ Student's t-test was used for the variables "age" and "body mass index," while the chi-squared test was applied to "White," "mixed," "primiparous," "secundiparous," "tertiparous or more," "vaginal," and "cesarean." The remaining variables were analyzed using Fisher's exact test. SD: standard deviation.

Prepartum complications such as premature rupture of the ovular membranes (PROM), decompensated diabetes mellitus, syphilis, hypertensive disorder, cervical insufficiency, trauma, polyhydramnios, cholestasis, 2nd–3rd-trimester vaginal bleeding, twin-to-twin transfusion syndrome (TTTS), preterm birth, fetal growth restriction (FGR), alloimmunization, and congenital infections were not different between the groups. In the pandemic group, six (12.0%) women were infected by COVID-19 infection during gestation, all after the first trimester. The prepartum complications are summarized in Table 2.

**Table 2:** Prepartum complications.

Prepartum complications	Pre-pandemia (n=38)	Pandemia (n=50)	p-value§
Any complications, n (%)	15 (39.5)	19 (38.0)	1.000
Premature rupture of ovular membranes, n (%)	3 (7.9)	1 (2.0)	0.311
Decompensated diabetes mellitus, n (%)	1 (2.6)	0 (0.0)	0.432
Syphilis, n (%)	1 (2.6)	0 (0.0)	0.432
Hypertensive disorder, n (%)	4 (10.5)	3 (6.0)	0.459
Cervical insufficiency, n (%)	3 (7.9)	1 (2.0)	0.311
Trauma, n (%)	1 (2.6)	0 (0.0)	0.432
Polyhydramnios, n (%)	1 (2.6)	0 (0.0)	0.432
Cholestasis, n (%)	1 (2.6)	0 (0.0)	0.432
Vaginal bleeding in the 2nd/3rd trimesters, n (%)	0 (0.0)	2 (4.0)	0.504
Twin-to-twin transfusion syndrome, n (%)	0 (0.0)	1 (2.0)	1.000
Preterm birth, n (%)	0 (0.0)	1 (2.0)	1.000
Fetal growth restriction, n (%)	0 (0.0)	1 (2.0)	1.000
Alloimmunization, n (%)	0 (0.0)	1 (2.0)	1.000
COVID-19 during pregnancy, n (%)	Not applicable	6 (12.0)	–
COVID-19 detected after the 1st trimester, n (%)	–	6 (100.0)	–
Urinary tract infection, n (%)	0 (0.0)	1 (2.0)	1.000
Other infection, n (%)	0 (0.0)	2 (4.0)	0.504

§ Chi-squared test was used for the variable "any complications." The remaining variables were analyzed using Fisher's exact test.

Similarly, fetal characteristics between the pre-pandemic and pandemic groups did not differ significantly. GA at death, sex, twinning, probable cause of death, and abnormal findings on ultrasonographic examination showed no statistically significant variations. Abnormal findings included malformations, FGR, Doppler alterations, polyhydramnios, oligohydramnios, placental hematoma, hydrops, gastroschisis, myelomeningocele, lower urinary tract obstruction (LUTO), congenital heart diseases, suspected aneuploidy, and others.

Among the causes of fetal death, congenital malformations were the most frequent in both groups. Maternal comorbidities, fetal disorders related to infection, and placental complications were present, and no significant differences were observed between the pre-pandemic and pandemic periods regarding these factors. The fetal characteristics are summarized in Table 3.

**Table 3:** Fetal characteristics.

Variables		Pre-pandemia (n=38)	Pandemia (n=50)	p-value§
Gestational age in weeks at death, mean (SD)		31.5 (6.4)	31.7 (5.9)	0.270
Intrapartum death, n (%)		2 (5.3)	3 (6.0)	1.000
	Female	11 (28.9)	17 (34.0)	0.785
	Male	15 (39.5)	14 (28.0)	0.365
	Unknown sex	12 (31.6)	19 (38.0)	0.690
Twin pregnancies		2 (5.3)	5 (10.0)	0.694
	Death of one fetus	1 (50.0)	2 (40.0)	1.000
	Death of two fetuses	1 (50.0)	3 (60.0)	0.631
Probable cause identified		21 (55.3)	26 (52.0)	0.930
	Decompensated diabetes mellitus	2 (5.3)	0 (0.0)	0.184
	Placental abruption	2 (5.3)	3 (6.0)	1.000
	Cervical insufficiency	2 (5.3)	0 (0.0)	0.184
	Congenital infection	2 (5.3)	1 (2.0)	0.576
	Malformation	9 (23.7)	14 (28.0)	0.832
	Hypertensive disorder	3 (7.9)	0 (0.0)	0.077
	Fetal growth restriction	2 (5.3)	2 (4.0)	1.000
	Premature rupture of ovular membranes	1 (2.6)	0 (0.0)	0.432
	Uterine rupture	0 (0.0)	1 (2.0)	1.000
	Twin-to-twin transfusion syndrome	0 (0.0)	1 (2.0)	1.000
	Other cause	0 (0.0)	4 (8.0)	0.130
Abnormal findings on ultrasound		27 (71.1)	31 (62.0)	0.509
	Malformation	9 (23.7)	14 (28.0)	0.832
	Fetal growth restriction	9 (23.7)	14 (28.0)	0.832
	Doppler abnormalities	1 (2.6)	4 (8.0)	0.384
	Polyhydramnios	5 (13.2)	1 (2.0)	0.081
	Oligohydramnios	9 (23.7)	13 (26.0)	1.000
	Placental hematoma	1 (2.6)	0 (0.0)	0.432
	Hydrops	3 (7.9)	6 (12.0)	0.726
	Twin-to-twin transfusion syndrome	0 (0.0)	1 (2.0)	1.000
	Gastroschisis	0 (0.0)	1 (2.0)	1.000
	Myelomeningocele	0 (0.0)	1 (2.0)	1.000
	Lower urinary tract obstruction	0 (0.0)	3 (6.0)	0.255
	Congenital heart diseases	1 (2.6)	8 (16.0)	0.072
	Suspected aneuploidies	8 (21.1)	9 (18.0)	0.931
	Other malformations	2 (5.3)	9 (18.0)	0.105

§ Student's t-test was applied to the variable "gestational age at death." Chi-squared test was applied to "female," "male," "unknown sex," "probable cause identified," "congenital malformation," "abnormal ultrasound findings," "fetal growth restriction," "oligohydramnios," and "suspected aneuploidies." The remaining variables were analyzed using Fisher's exact test. SD: standard deviation.

## DISCUSSION

In this cohort study, no significant differences were observed between the pre-pandemic and pandemic groups in terms of maternal demographics, pregnancy characteristics, or prepartum complications. Factors such as maternal age, BMI, parity, mode of delivery, comorbidities (e.g., diabetes mellitus, chronic hypertension, and preeclampsia), and complications like PROM, cholestasis, TTTS, and FGR were similar. Fetal characteristics, including GA at death, probable causes, and abnormal ultrasonographic findings (e.g., malformations, hydrops, and congenital heart diseases), also showed no significant variation between the groups.

The impact of the COVID-19 pandemic on fetal death statistics, including stillbirth and neonatal mortality rates, has been investigated across diverse geographic and healthcare settings, revealing considerable variability in findings. For instance, a study conducted in Alabama, USA, found no significant changes in stillbirth rates during the initial and delta phases of the pandemic compared to the baseline period. Interestingly, a decline in neonatal mortality rates was observed during the pandemic period in this region^7^. In contrast, research from Alberta, Canada, identified a decrease in perinatal death rates as pandemic exposure increased^8^. This trend was particularly pronounced among pregnancies with high antepartum risk scores, suggesting that government response programs and specific pandemic control measures might have positively ­influenced these outcomes^9^.

Conversely, data from Scandinavia (Sweden, Denmark, and Norway) revealed no significant changes in the risk of fetal death, including miscarriages and stillbirths, following the implementation of COVID-19 mitigation measures^10^. Similarly, in Ontario, Canada, researchers found no unusual variations in stillbirth or preterm birth rates during the first year of the pandemic compared to the preceding 17.5 years^11^. In contrast, a study in Nigeria reported a significant increase in stillbirths and newborn deaths during the pandemic^12^.

These findings collectively illustrate the heterogeneous impact of the COVID-19 pandemic on fetal death outcomes, strongly influenced by regional differences in healthcare system resilience and accessibility. In the context of Brazil, a country characterized by immense geographic diversity and significant social disparities, outcomes may vary considerably across ­different regions. As our study demonstrates when analyzed in light of the literature, the pandemic's impact on fetal and neonatal outcomes in Brazil likely mirrors this variability, underscoring the need for region-specific data and tailored interventions.

We did not find studies in the literature reporting specific data on fetal mortality before and during the pandemic. Existing studies primarily focus on the infection itself, associating worse outcomes with COVID-19 infection in pregnant women. A study conducted in the state of Bahia, Brazil, examined the relationship between acute respiratory distress syndrome (ARDS) during pregnancy and fetal death in the context of the pandemic^13^. This research revealed that women experiencing ARD, regardless of etiology, faced a substantially heightened risk of fetal death. Another cohort study using data from the public healthcare system in Brazil explored the broader impact of COVID-19 on maternal and fetal mortality, a finding that pregnant women with confirmed severe acute respiratory syndrome-coronavirus-2 (SARS-CoV-2) infection were nearly twice as likely to experience fetal death or stillbirths compared to uninfected women^14^. Complementing these findings, another Brazilian study reported an association between SARS-CoV-2 infection and adverse outcomes such as stillbirths and preterm births, even among mildly symptomatic pregnant women^15^.

In contrast to these findings, our study, conducted in a private maternity hospital in Rio de Janeiro—a well-equipped and resource-rich healthcare center—found no significant differences in fetal mortality rates before and during the COVID-19 pandemic. This outcome likely reflects the robust healthcare infrastructure and quality of care available in such settings. However, this scenario is not representative of the majority of healthcare facilities in Brazil, which often face significant resource constraints. The disparities between well-equipped private centers and the under-resourced public healthcare system underscore the critical role of socioeconomic and structural factors in determining health outcomes during the pandemic. While our findings suggest that well-resourced environments can mitigate some of the adverse effects of global health crises, the broader reality for most of Brazil's population remains starkly different, as highlighted by the literature. Large-scale, multicentric studies comparing different healthcare settings could provide a more comprehensive understanding of the impact of the pandemic on fetal deaths.

This study has several limitations that should be ­acknowledged. First, it was conducted in a single, private maternity hospital in Rio de Janeiro, which may not be representative of the broader Brazilian healthcare context, particularly public and resource-limited settings. Second, the sample size was relatively small, potentially limiting the statistical power to detect subtle differences between the pre-pandemic and pandemic groups. Additionally, as a retrospective cohort study, it is subject to inherent biases, including potential inaccuracies in medical records and the inability to control for unmeasured confounding variables. These limitations highlight the need for caution in generalizing the findings and underscore the importance of further research involving diverse populations and healthcare settings.

## CONCLUSION

While this study found no significant differences in fetal death rates before and during the COVID-19 pandemic in a well-equipped private maternity hospital, it is crucial to recognize that this scenario is not representative of the majority of healthcare facilities in Brazil. The observed stability in fetal mortality rates likely reflects the robustness of private healthcare infrastructure, emphasizing the need for targeted strategies to address healthcare disparities and enhance maternal–fetal care in resource-limited settings. Further studies with larger, more diverse populations are necessary to develop equitable public health policies and ensure better outcomes for all pregnant individuals, particularly during global health crises.

## Data Availability

The datasets generated and/or analyzed during the current study are available from the corresponding author upon reasonable request.
